# Solvent-Free Synthesis of Iron-Based Metal-Organic Frameworks (MOFs) as Slow-Release Fertilizers

**DOI:** 10.3390/polym13040561

**Published:** 2021-02-13

**Authors:** Yaxiao Du, Xuebin Xu, Fei Ma, Changwen Du

**Affiliations:** 1 The State Key Laboratory of Soil and Sustainable Agriculture, Institute of Soil Science Chinese Academy of Sciences, Nanjing 210008, China; duyaxiao@foxmail.com (Y.D.); xuxuebin@issas.ac.cn (X.X.); fma@issas.ac.cn (F.M.); 2 College of Advanced Agricultural Sciences, University of Chinese Academy of Sciences, Beijing 100049, China

**Keywords:** mechanochemical synthesis, slow release fertilizer, metal-organic frameworks, urea

## Abstract

Metal-organic frameworks (MOFs) were usually synthesized in hydrothermal conditions; in this study, a more energy-saving, easier to control, and solvent-free mechanochemical method was firstly applied to synthesize MOFs with varied reactants as slow release fertilizer, and the components and structures were characterized by X-ray diffraction (XRD), Fourier transform infrared total attenuated reflectance (FTIR-ATR), and laser-induced breakdown spectroscopy (LIBS). Results showed that three MOFs (compounds I, II, and III) were obtained, the MOFs were confirmed as oxalate phosphate oxalate frameworks (OPA-MOF), and ions were adsorbed between layers that contributed to the contents, while urea molecules mainly impacted the structure. The elemental compositions significantly varied among the three compounds; compound I showed the highest content of N (4.91%), P (15.71%), and Fe (18.60%), compound III indicated the highest content of C (6.52%) and K (12.59%), while the contents of C, K, P, and Fe in compound II were in the medium range. Similar release profiles of Fe and P were found among the three MOFs, and the release rates of nutrients were demonstrated as the order of N > K > P > Fe. The compositions and release profiles demonstrated potential application of MOFs as a novel slow-release fertilizer.

## 1. Introduction

With the growth of the world population, food security has become more and more important. How to maintain the harmony and balance between agricultural production, soil, and the environment is a topic of concern. Many problems of soil and environment were brought by continuous large-scale grain production due to a large amount of fertilizer input, which resulted in a shortage of trace elements [1], soil degradation [2], groundwater, and air pollution [3]. How to improve the use efficiency of fertilizer was the core problem in fertilization, and slow-release fertilizer provided an option regarding this issue. The release rates of nutrients were delayed or controlled in slow release fertilizer to well match the absorptions of crops for reaching higher use efficiency. However, there were still some weakness in conventional slow/controlled release fertilizers in application; for example, the potential negative impact of membrane material or carrier residue, insufficient initial nutrient release rate, the unstable quality of nutrients release, and high synthesis and processing costs [4]. The unique properties of metal-organic frameworks (MOFs), such as various ligands, high porosity, and a flexible and adjustable structure [5], provided novel carriers for the slow release of nutrients.

Although currently MOFs demonstrate broad application, very little attention has been paid in agriculture. In 2016, Anstoetz et al. synthesized and characterized an oxalate phosphate amine metal organic framework (OPA-MOF) using phosphoric acid, ferric chloride, and oxalic acid as substrates and urea as an organic template by the hydrothermal method [6], and it was first applied as a slow-release fertilizer in a wheat pot experiment, which showed that OPA-MOF effectively improved nitrogen-use efficiency through a slow release of ammonium in alkalizing soil [7]. The highest nitrogen content of the MOF product was 8.2% [8]. In 2019, Wu et al. synthesized OPA-MOF with a similar method [9], and the nutrient release performance and degradation process were further explored in a rice field, which showed that the collapse of the OPA-MOF structure controlled the slow release of NH_4_^+^ and improved the agronomic traits, rice yield, nitrogen-use efficiency, and nitrogen content in soil [10]. These studies preliminarily confirmed the potential application of OPA-MOF as a slow-release fertilizer; however, the hydrothermal method with urea as structure-directing agent (SDA) was conventionally used, which caused urea decomposition, required high temperature, generated huge autogenous pressure, and took a long time in the synthesis and drying process. As a result, the cost of synthesis and processing was still too high as a fertilizer, which limited the application in agriculture.

Mechanochemical synthesis, i.e., the chemical synthesis reaction initiated or maintained by the direct absorption of mechanical energy [11,12], took on the advantages of easy mass production, high quantitative yield, low heat, and avoiding a large amount of solvent [13], which provided new ideas for the synthesis of MOFs. One of the most important technologies in mechanochemical synthesis was solvent-free synthesis that uses hydrated metal salts as raw materials. The coordination water was released after grinding [14]. The first report on the solvent-free synthesis of MOFs was in 2006, Pichon et al. obtained [Cu(INA)_2_], a porous structure, by grinding copper acetate and isonicotinic acid together and then heating the raw material to remove water and acetic acid. This method has higher efficiency in material, energy, and time [15]. In recent years, Fe, Zn, Cu, Co, Ca, and various rare earth elements were used as metal nodes to synthesize MOFs and tried as applications in pollutant adsorption and drug release [16,17,18,19,20].

In this study, the solvent-free mechanochemical method was firstly applied to synthesize MOFs as a slow-release fertilizer. Three iron phosphate oxalic acid frameworks were synthesized and characterized, and the nutrient content, product structure, and nutrient release behavior were detected for exploring the feasibility of MOFs as slow-release fertilizer.

## 2. Materials and Methods

### 2.1. Materials

Ferric chloride (FeCl_3_·6H_2_O), dipotassium hydrogen phosphate (K_2_HPO_4_·3H_2_O), diammonium hydrogen phosphate ((NH_4_)_2_HPO_4_), oxalic acid (H_2_C_2_O_4_·2H_2_O), and urea (CH_4_N_2_O) (analytical grade) were purchased from Nanjing Ronghua Scientific Equipment Co., Ltd. (Jiangsu, China). Reactants were crushed to a fine powder using the planetary micro mill (Puluerisette 7, FRISCH, Idar-Oberstein, Germany) for use.

### 2.2. Synthesis of MOFs

Reactants and substrate ratios (I, II, and III) were set as the following: I, 1 FeCl_3_·6H_2_O, 2 (NH_4_)_2_HPO_4_, 1 H_2_C_2_O_4_·2H_2_O; II, 1 FeCl_3_·6H_2_O, 2 K_2_HPO_4_·3H_2_O, 1 H_2_C_2_O_4_·2H_2_O; III, 1 FeCl_3_·6H_2_O, 2 K_2_HPO_4_·3H_2_O, 1 H_2_C_2_O_4_·2H_2_O, 2 CH_4_N_2_O. Substrates were put into the agate grinding tank and ground using the planetary micro mill at 600 r/min speed for 12 min. Then, they were heated at 333 K for 10 h. MOFs were synthesized accordingly as compound I, II, and III, and were washed, filtered, and dried for analysis.

### 2.3. Element Composition

The contents of C, N, H, and O of the three MOFs were determined by an element analyzer (vario EL cube, Elementar, Heraeus, Germany). The compound was completely dissolved with HCl (6 mol/L), and P, Fe, andK were determined by inductively coupled plasma optical emission spectrometer (ICP-OES) (Thermo Fisher Scientific, Waltham, MA, USA). Ammonium nitrogen and urea were qualified by indophenol blue colorimetry and para-dimethyl-amino-benzaldehyde (PDAB) colorimetry.

### 2.4. Spectral Characterization

X-ray diffraction (XRD) spectrum of the compounds was collected on a X-ray diffractometer (Model XRD-7000, Shimadzu Corporation, Kyoto, Japan) over the 2θ range of 2–60° by using Cu Kα radiation at a 0.02° step size and a 5°/min scanning rate. Jade 6 (MDI, USA) was used to smooth, fit the spectrum, remove the amorphous peaks, and calculate the crystallinity. Fourier transform infrared total attenuated reflectance (FTIR-ATR) spectra were scanned on a handheld TruDefender Fourier transform spectrometer (Thermo Fisher Scientific, Waltham, MA, USA). Spectra were acquired in the range of 4000–650 cm^−1^ with a spectral resolution of 4 cm^−1^. Diamond was used as the ATR-reflecting element for improved contact, and a blank reference was scanned before each sample was scanned. The background was subtracted from each scan to correct for atmospheric and instrumental noise. A MobiLIBS system (IVEA Solution, Paris, France) was used for laser-induced breakdown spectroscopy (LIBS) to determine the atomic composition and content of the MOFs. The system consisted of a fourth-harmonic Nd:YAG laser (Quantel, Paris, France) driving 5-ns pulses. The frequency, delivery energy, and wavelength of the pulsed laser were 20 Hz, 16 mJ, and 266 nm (Nd-YAG), respectively, and 2× 2 matrices were set for each shot.

### 2.5. Principal Component Analysis

The *Mapstd* and *Repmat* function in Matlab (R2018a, the Math Works, Natick, MA, USA) was used to standardize and combine the FTIR-ATR spectra and LIBS spectra, and then principal component analysis was performed based on the fused spectra.

### 2.6. Nutrient Release Behavior

First, 1.5 g of MOFs was accurately weighed and placed in a reagent bottle containing 50 mL of deionized water and cultured at 298 K at constant temperature for three weeks, and the nutrients (N, Fe, K, P) in the solution were determined every week.

## 3. Results

### 3.1. Elemental Analysis

The product yields and element contents of the three MOFs are shown in Table 1. The direct yields of the three MOF products were similar, which ranged from 33% to 34%, which was high enough for practical production. The N content in compound I was the highest as 4.91%, which existed in the form of NH_4_^+^, which was not from the hydrolysis of urea, since there was no urea input in the substrates [7,21,22]. Although some amount of urea was involved in the reaction III, the N content was only 0.11%, which meant that urea was difficult to load into the MOF, and most of the urea was lost. When K_2_HPO_4_·3H_2_O was used instead of (NH_4_)_2_HPO_4_, the K contents of compounds II and III are 12.59% and 11.48%, respectively, which meant that ions were easily loaded into MOF, while it was difficult to load molecules. Compound II showed the highest C content and the lowest P and Fe content.

As compound fertilizer, the nutrients content should be more than 20%, and compounds I, II, and III reached 40.89%, 45.97%, and 41.78%, respectively, which could be identified as compound fertilizer with high nutrients content (>40%). Since Fe is also the necessary nutrient for crops, the nutrients content was even much higher if considering the micronutrients.

### 3.2. X-ray Powder Diffraction

**Figure 1:**
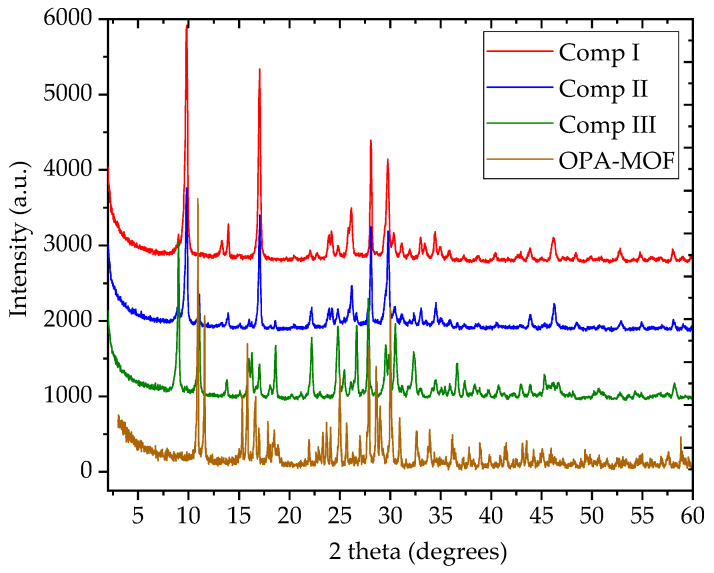
X-ray powder diffraction spectra of MOFs (compounds I, II, and III) and reference oxalate phosphate amine metal organic framework (OPA-MOF) [10].

The X-ray powder diffraction spectra of compounds I, II, and III were compared with reference OPA-MOF [10] (Figure 1) and the data of the international diffraction data center. It is found that the three compounds were new phases with crystallinity of 92.28%, 96.72%, and 93.97%, respectively. It was observed that all reactants participated in the reaction, and the compounds crystallized well. Combined with the results of element contents, compounds I and II, which use NH_4_^+^ and K^+^ as counter ions, respectively, have similar crystal structures, while the crystal structure of compound III was obviously changed due to the addition of urea input in the substrate, which meant that urea’s function was related with the compound structure rather than the N content.

### 3.3. FTIR-ATR Characterization

**Figure 2:**
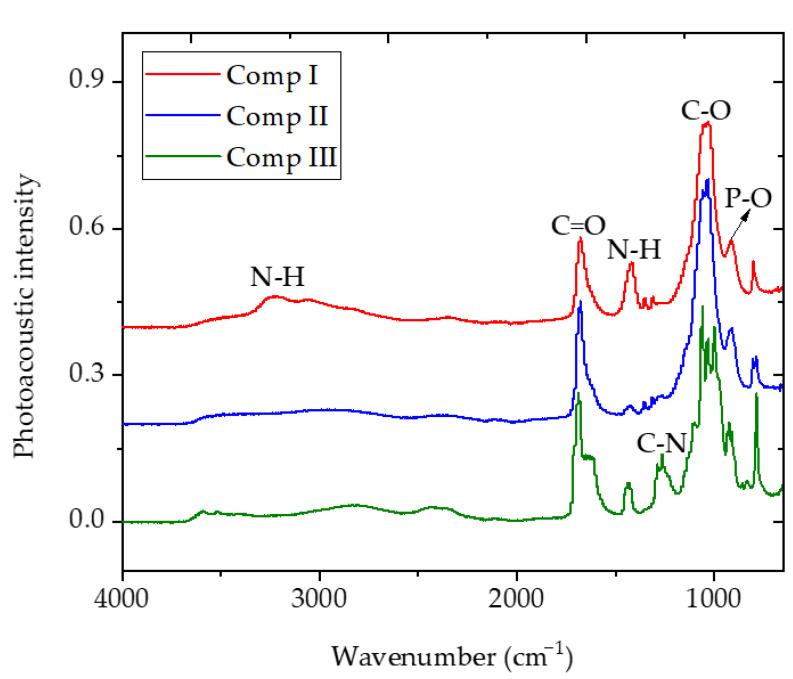
Fourier transform infrared total attenuated reflectance (FTIR-ATR) spectra of MOFs (compounds I, II, and III).

The FTIR-ATR spectra of MOFs are shown in Figure 2. The absorption bands for compound I were obtained at 802, 916, 1030, 1312, 1353, 1418, 1683, and 3226 cm^−1^. The absorption peaks at 916, 1030, and 1683 cm^−1^ were associated with P-O, C-O, and C = O, while 1418 and 3226 cm^−1^ were associated with the stretching vibration of N-H. When K_2_HPO_4_·3H_2_O was used instead of (NH_4_)_2_HPO_4_ in compound II, the absorption peak of N-H disappeared, while other peaks remained almost unchanged, which indicated that the substitution of K^+^ for NH_4_^+^ produced a similar molecular structure. Compound III with urea as a nitrogen source showed obvious absorption peaks of acylamide (1615–1665, 1265 cm^−1^), which indicated that compound III contained a small amount of urea rather than ammonium, which was consistent with the results of element analysis.

### 3.4. LIBS Characterization

**Figure 3:**
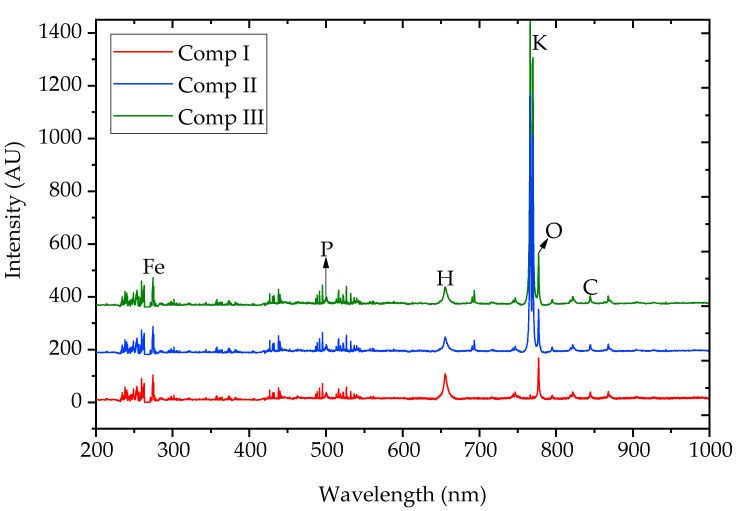
Laser-induced breakdown spectroscopy (LIBS) spectra of MOFs (compounds I, II, and III).

The LIBS spectra of MOFs are shown in Figure 3. According to the standard atomic spectrum data provided by the National Institute of standards and Technology (NIST), the atomic spectral lines of Fe (274.6 nm), P (500.3 nm), H (655.6 nm), O (777.3 nm), and C (844.8 nm) were found in compound I, and strong characteristic spectral lines of K (766.5 nm, 769.9 nm) appeared in products II and III. The spectral line of N might be disturbed by nitrogen in the air and was difficult to identify. When (NH_4_)_2_HPO_4_ was replaced by K_2_HPO_4_·3H_2_O, a sharp K line (around 767 nm) was observed, while no significant effects were found for the input of urea, which further verified that urea functioned in the formation of MOF structure while it demonstrated little contribution in N loading.

### 3.5. Principal Component Analysis of Fused Spectra

**Figure 4:**
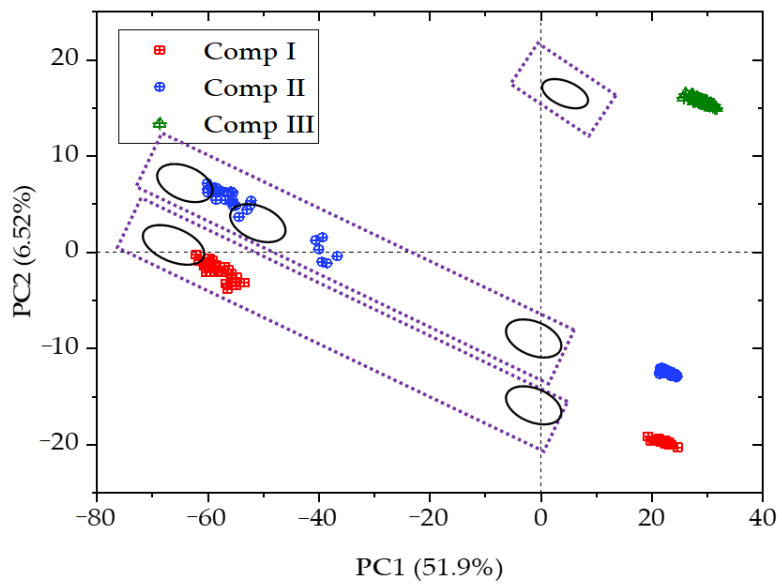
Fused spectra-based principal component analysis.

For further identification of the synthesized MOFs, the LIBS and FTIR-ATR spectra of the MOFs (compounds I, II, and III) were fused, including the standardization and connection of the spectra data, and then, principal component analysis was performed (Figure 4). The MOFs could be well identified though the PCA distribution. A much more close distribution was observed for compound III, and both the distributions for compound I and II were far from that of compound III; and there was only one spot for compound III, while there were two and three spots for compounds I and II, respectively, and compounds I and II were very similar.

### 3.6. Nutrient Release Profile

**Figure 5:**
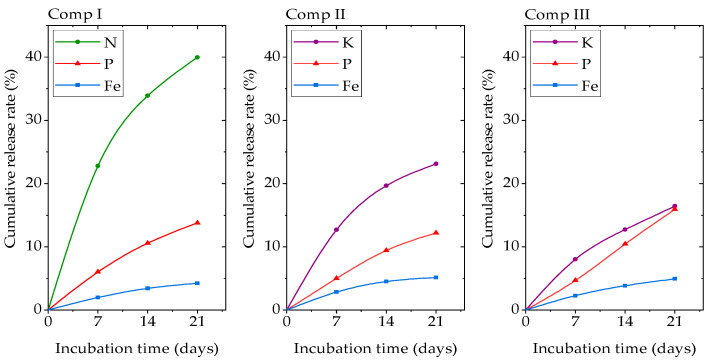
Nutrient release behaviors of MOFs (compound I, II, and III).

The nutrient cumulative release curves of the MOFs are shown in Figure 5. The cumulative release rates of N of compound I at 7 d, 14 d, and 21 d were 22.78%, 33.9%, and 39.96%, respectively. The cumulative release rates of K of compound II and compound III at 21d were 23.12% and 16.42%, respectively. The cumulative release rates of P of compound I, II, and III at 21d were 13.76%, 12.18%, and 15.92%, respectively, and for Fe, they were 4.25%, 5.12%, and 4.92%, respectively. In comparison, the cumulative release rates of N, P, and Fe in OPA-MOF synthesized by the hydrothermal method were 5.25%, 4.06%, and 3.48%, respectively [23]. The nutrient release rates of the three compounds synthesized by the mechanochemical method were faster, which might be related to the low heat condition of the reactions.

As the main elements of the iron phosphate layer in the MOF, P and Fe release rates were much slower, which were decided by the collapse of the frameworks; thus, the P and Fe release rates for the three MOFs were similar, while the release of NH_4_^+^ or K^+^ in the interlayer as counter ions was much faster (Figure 5). Although NH_4_^+^ and K^+^ showed the similar ionic radius and the same positive charge, the release rate of NH_4_^+^ was significantly faster than that of K^+^, and urea input further slowed the release rate of K^+^.

## 4. Discussion

### 4.1. Structure of MOFs

**Figure 6:**
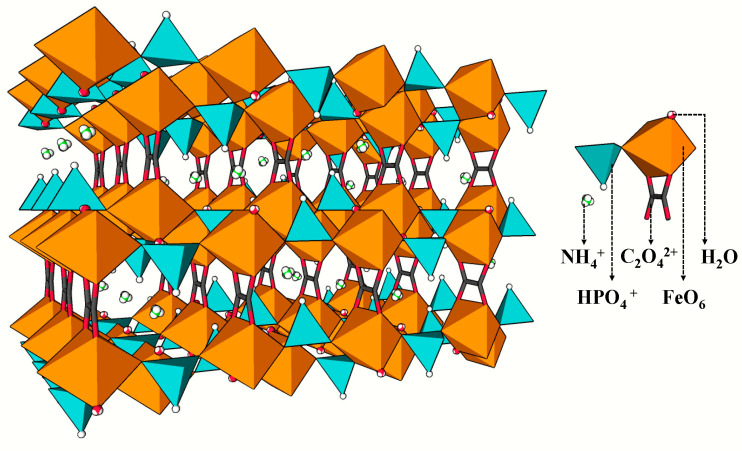
Structure diagram of compound I. FeO_6_, octahedra yellow; PO_4_, tetrahedra blue; O, red; C, black; N, green; H, white.

Based on the above experimental results, it was confirmed that the compounds were iron phosphate oxalate frameworks. According to the previous studies [24,25,26,27,28,29,30,31], the frameworks were generally composed of an iron phosphate layer and oxalic acid ligand, and ions, water molecules, or other templates are adsorbed between layers by Coulomb force, Van der Waals forces, and hydrogen bonds. The total framework is shown in Figure 6.

In compound II, when potassium ions are used as substrates, potassium ions replace the interlayer ammonium ions in compound I, and they play a similar role in the structure formation process. In reaction III, about 14% of urea might be decomposed during grinding or heating, and the remaining urea did not load into the frameworks. Although there was around 14% decomposed urea, most of the hydrolyzed ammonium also did not load into the framework, which might be due to the lag of ammonium as a substrate after the frameworks formation. However, urea showed significant effects on the frameworks formation of the compound as a structure-directing agent [32]; for example, the ratios of C/P and C/Fe changed, which indicated that the coordination mode of Fe^3+^ might have changed, resulting in the change of the structure of the iron phosphate layer. However, the molecular mechanism of how urea affected the formation of the frameworks remained unclear.

### 4.2. MOFs as Fertilizers

In the three mixtures obtained by reactions I, II, and III, the contents of residual reactants were 63.2%, 63.56%, and 59.51%, respectively, which could be used as available nutrients to supplement the needs of crops in the initial stage; i.e., the recovery of residual reactants and the secondary drying of compounds could be avoided, and the mixture of the MOF products could be directly used without further treatment such as separation, which would well match with crop requirements while significantly decreasing the production cost. It was worth noting that MOF materials often use Fe, Zn, and Cu as metal centers, which might bring the risk of soil heavy metal pollution. Therefore, reasonable application according to crop requirement should be considered.

Although the hydrated radius of K^+^ and NH_4_^+^ was the same (around 0.331 nm), the radius of crystalized K^+^ (0.133 nm) was smaller than that of crystalized NH_4_^+^ (1.48 nm). Due to more complete hydration in compound II, the practical radius of K^+^ in the frameworks might be larger than the practical radius of NH_4_^+^ in compound I, and urea addition might decrease the distance between layers in frameworks [8], which resulted in a lower release rate of K^+^ in compound III. It was found that K^+^ induced the formation of doubly inter-penetrating frameworks, participated in metal coordination, and then enhanced the stability of an MOF [32], which might be another reason for the slower release rate of K^+^. The nutrient release rate was faster than the MOF compounds obtained by the hydrothermal method [23], which might be due to the fact that metastable compounds were produced in solid state as low heat reactions, resulting in weak water stability or thermal stability [8]. How to enhance the water stability of solid phase synthesis products was worth exploring in further study.

At present, the slow/controlled release fertilizers in the market are mainly coated fertilizers, which are composed of an internal fertilizer and a membrane material. The release of nutrients is controlled by an insoluble or hydrophobic coating. MOF fertilizers were directly synthesized from reactants containing N, K, P, and Fe, and the cost of the mechanochemical method was greatly reduced compared with that of the hydrothermal method or coated method; the cost was about 500 $/ton, which was acceptable in application, since the costs of most commercial coated NPK controlled release fertilizer are around 1000 $/ton. Furthermore, it is still possible to reduce the cost by optimizing or selecting lower cost organic ligands or metal salts.

## 5. Conclusions

Iron phosphate oxalate frameworks were successfully synthesized at lower temperature through the mechanochemical method, and they showed similar element composition and structure with the products obtained by hydrothermal synthesis. P and Fe were loaded into MOF as the structural elements, and NH_4_^+^ and K^+^ were loaded into the layers among the MOF frameworks, while urea addition mainly functioned in the structure formation of MOF. The total nutrients contents of the synthesized MOFs were higher than 40%, which could be applied as high-grade compound fertilizer. P, Fe, K^+^, and NH_4_^+^ were slow released, and as structural elements, the release rates of P and Fe were relatively lower, while the release rates of K^+^ and NH_4_^+^ were faster; the release rates of K^+^ was lower than that of NH_4_^+^, and urea addition further delayed the release of K^+^. Therefore, a solvent-free mechanochemical method could be applied to synthesize MOFs with high nutrients content and good release behavior as well as a much lower production cost, which significantly improved the application potential of MOFs as slow-release fertilizer.

## Figures and Tables

**Table 1:** Elemental compositions of synthesized metal-organic frameworks (MOFs) as well as the production yields (%).

	N	K	C	H	P	Fe	Yield	Nutrients
Comp I	4.91	0	4.53	3.60	15.71	18.60	33.21	40.89
Comp II	0	11.48	4.75	1.66	14.16	16.22	33.93	45.97
Comp III	0.11	12.59	6.52	2.05	11.76	15.45	33.79	41.78

Note: Nutrients refer to the macronutrients content in fertilizer, which was conventionally calculated as the percentage content of N + K_2_O + P_2_O_5_ rather than N + K + P.

## Data Availability

Data is contained within the article.
